# Fast X-Ray Fluorescence Microtomography of Hydrated Biological Samples

**DOI:** 10.1371/journal.pone.0020626

**Published:** 2011-06-02

**Authors:** Enzo Lombi, Martin D. de Jonge, Erica Donner, Peter M. Kopittke, Daryl L. Howard, Robin Kirkham, Chris G. Ryan, David Paterson

**Affiliations:** 1 Centre for Environmental Risk Assessment and Remediation, University of South Australia, Mawson Lakes, South Australia, Australia; 2 CRC CARE, Salisbury, South Australia, Australia; 3 X-Ray Fluorescence Microscopy, Australian Synchrotron, Clayton, Victoria, Australia; 4 School of Agriculture and Food Sciences, The University of Queensland, St. Lucia, Queensland, Australia; 5 Materials Science and Engineering, Commonwealth Scientific and Industrial Research Organisation (CSIRO), Clayton, Victoria, Australia; 6 Earth Science and Resource Engineering, Commonwealth Scientific and Industrial Research Organisation (CSIRO), Clayton, Victoria, Australia; George Mason University, United States of America

## Abstract

Metals and metalloids play a key role in plant and other biological systems as some of them are essential to living organisms and all can be toxic at high concentrations. It is therefore important to understand how they are accumulated, complexed and transported within plants. In situ imaging of metal distribution at physiological relevant concentrations in highly hydrated biological systems is technically challenging. In the case of roots, this is mainly due to the possibility of artifacts arising during sample preparation such as cross sectioning. Synchrotron x-ray fluorescence microtomography has been used to obtain virtual cross sections of elemental distributions. However, traditionally this technique requires long data acquisition times. This has prohibited its application to highly hydrated biological samples which suffer both radiation damage and dehydration during extended analysis. However, recent advances in fast detectors coupled with powerful data acquisition approaches and suitable sample preparation methods can circumvent this problem. We demonstrate the heightened potential of this technique by imaging the distribution of nickel and zinc in hydrated plant roots. Although 3D tomography was still impeded by radiation damage, we successfully collected 2D tomograms of hydrated plant roots exposed to environmentally relevant metal concentrations for short periods of time. To our knowledge, this is the first published example of the possibilities offered by a new generation of fast fluorescence detectors to investigate metal and metalloid distribution in radiation-sensitive, biological samples.

## Introduction

The uptake of metal(loid)s, whether acting as micronutrients or contaminants, is a process of major importance in biological systems. Molecular biology techniques such as fluorescent protein tagging and immunohistological staining allow the expression of key metal(loid) transporters to be imaged [e.g. 1]. Yet in order to assess the function of candidate transporters, the localisation of the metal(loid)s within the relevant tissue must also be determined. The analytical challenges of imaging low metal(loid) concentrations in a biologically relevant state are considerable, especially when the specimen is susceptible to radiation damage [for review see 2]. In the case of plant roots investigated here, the highly-hydrated nature of the root poses a challenge due to dehydration, and also compounds the issue of radiation damage due to the potential mobility of metal(loid)s in aqueous environments. Approaches using spectroscopic techniques such as scanning electron microscopy coupled with energy dispersive x-ray detection (SEM-EDX), particle induced x-ray emission (PIXE) or secondary ion mass spectrometry (SIMS) require considerable sample preparation in order to obtain cross sections for analysis. This is because all these techniques are inherently surface sensitive, and determining the distribution of elements across root tissues therefore requires the analysis of cross sections. Dehydration of the sample, and often resin-embedding, is generally required before or after sectioning. However, such sample preparation procedures have the potential to introduce artifacts due to metal(loid) redistribution [e.g. 3]. An exception could be provided by cryo-stage spectroscopic techniques such as cryo-SEM-EDX [e.g. 4] but although these minimise the risk of artifacts they also generally lack the necessary sensitivity due to increased bremsstrahlung background.

Synchrotron-based x-ray fluorescence microtomography can image metal(loid) distributions with high sensitivity and requires minimal specimen preparation thanks to the penetrating nature of hard x-rays [5]. In order to image elemental distributions in cross section the specimen is scanned along a transect. The sample is then rotated by a small angle and the process repeated numerous times to obtain sinograms, the raw input data for tomographic analyses. A 2D ‘virtual’ cross section can then be reconstructed using any one of a number of algorithms [6]. Multiple elements are imaged simultaneously. However, this has traditionally required significant data acquisition time. Bleuet et al. [7] recently indicated that collection of one such virtual cross-section for a 200-µm diameter object assuming 90 projections, a 1 µm scanning step and a typical dwell of 1 s per pixel would require at least 5 hours (not including overhead times due to stage motion and detector read-out). While this analysis time has not proven prohibitive for plant parts inherently low in water content such as seeds [8], [9], the situation is different in the case of highly hydrated samples such as plants roots. In this case, the samples are in fact likely to suffer from both radiation damage and changes in shape caused by dehydration. As a consequence, studies conducted in plants using these techniques have employed dried samples. This is the case for the study conducted by Blute et al. [10] who used this technique to assess the distribution and speciation of As associated with Fe plaques in the roots of *Typha latifolia* (cattail). Likewise, freeze dried specimens were used by McNear et al. [11] to investigated the distribution of Ni in *Alyssum murale*. In this case the authors pointed out that ‘attempts were made to image “fresh” plant tissues using fluorescent CMT; however, it was found that shock freezing and partial drying was required because the high power density of the microfocused x-ray beam caused motion associated with dehydration in “live” *Alyssum* plant tissue which compromised reconstruction of the tomograms’ McNear et al. [11].

Recent advantages in fluorescence detection technology have the potential to overcome these limitations due to vastly improve acquisition performance [12]. Recently, we have demonstrated that, using the recently developed Maia detector, megapixel elemental images of *Ordeum vulgare* (barley) grains could be collected in comparable times as for conventional detector technologies but with lateral definition improved approximately 100 times (as compared to previous synchrotron studies [e.g. 13]). The Maia detector system is a new generation of x-ray fluorescence detectors developed jointly by CSIRO (Australia) and BNL (USA). Maia uses an annular array of 384 silicon-diode detectors positioned in a backscatter geometry to subtend a large (∼1.3 sr) solid-angle and to achieve high count-rate capacity [14]. Maia is designed for ‘on-the-fly’ (continuous) scanning, using a nuclear physics approach to data acquisition that results in essentially zero overheads. Consequently, transit times per pixel can be as short as ∼50 µs with a count rate capacity greater than 10 M/s [15].

In this study we used the Maia to assess the distribution of two widespread metal contaminants in plant roots. A number of approaches were tested and we report, to our knowledge for the first time, the distribution of Ni and Zn across hydrated plant roots exposed to environmentally relevant metal concentrations for short periods of time.

## Results and Discussion

**Figure 1:**
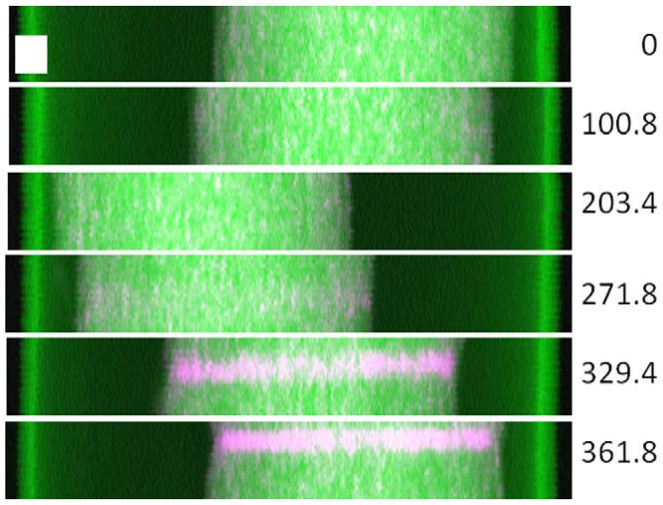
Zinc (magenta) x-ray fluorescence maps of the same *V. unguiculata* root volume collected at 6 orientations. The Compton signal is in green. The scale box is 50 µm wide by 20 µm tall. The onset of beam-induced damage can be seen from the maps collected at 271.8° onwards (all 100 frames available as **Supplementary Movie 1** online).

Initially we attempted to collect 3D tomograms by acquiring a series of 2D maps over 100 angles spaced over 360°. A full 360-degree range was chosen to probe self-absorption effects, but measurements over the second half of the range were made unique by offsetting them by 1.8-degrees relative to the first. The sampling interval was 2×2 µm with a transit time of 2.6 ms. A complete series of 2D projections required ca. 5 hours. Six of these projections are reported in Fig. 1, where the onset of beam damage is evident after approximately 75 projections (271.8°). The damage was revealed by the appearance of a concentrated band of Zn (magenta) which became increasingly marked as the acquisition progressed. This structure was initially not observed in the Compton signal (green) indicating no significant mass-loss [16] or light-element (H, C, N, O) redistribution. This indicates that the x-ray beam is most likely the key factor responsible for the observed damage rather than dehydration of the sample during analysis. However, by the end of the tomographic series mass loss, and consequently loss of structural integrity of the root, is also apparent, as a restriction in correspondence of the area of Zn accumulation is also evident. Tomographic reconstruction using the first 50 projections, which did not appear to suffer from beam-induced damage, showed that metal distribution was not well resolved, consistent with insufficient angular sampling (see Supplementary Movie 2). It should be noted here, that only a small portion of the root volume was analyzed (40 µm in the vertical direction). As beam damaged occurred even when such a limited volume was investigated, we abandoned the idea of collecting 3D tomograms as exploring a smaller volume would have produced information not very dissimilar from what can be obtained from 2D tomograms.

**Figure 2:**
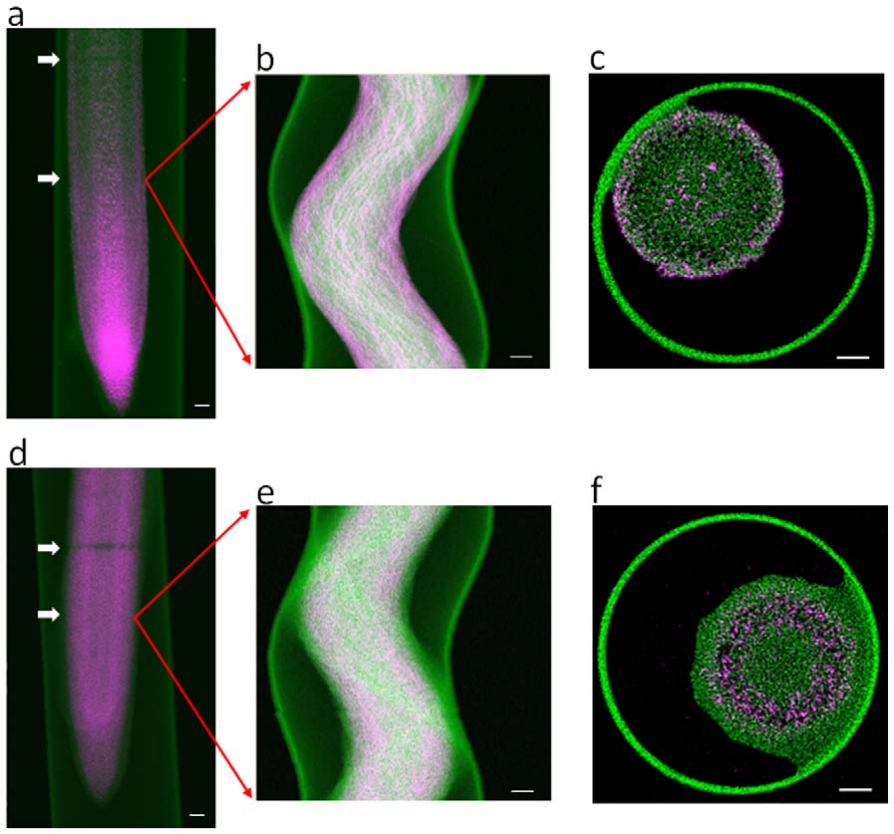
2D maps for Zn (a) and Ni (d) are reported in magenta; the intensity of the signal is proportional to the intensity of the colour. The Compton signal is in green. Scans of 200 rotations acquired over 360° were used to generate the sinograms and tomographic reconstructions for Zn (**b & c**) and Ni (**e & f**). White scale bars are 100 µm.

Therefore, in order to reduce beam damage we resorted to the acquisition of 2D virtual cross sections. Although single slice tomography does not reduce the local dose delivered to the specimen, it reduces the secondary exposure, as well as the total time required for data acquisition. Furthermore, as the root cells are typically around 20–100 µm long, this approach substantially reduces the total dose delivered to each cell in comparison to the 3D approach above. A single transect was scanned as the sample was rotated from 0° to 360° in 200 steps. A full rotation was used so as to enable assessment of beam damage and self absorption by the specimen. The sampling interval was 2 µm and two transit times were tested on each root (7.8 ms and 3.9 ms, with corresponding acquisition times of about 14 and 9 min). The analyses were conducted at a distance of ca. 1.5 mm to 2.0 mm from the root tip. Results are reported in Fig. 2. The 2D elemental maps, collected after the tomographic data, show the distributions of Zn (upper) and Ni (lower) and the Compton signal, which indicates the distribution of the lighter elements and thereby the location of the capillary and root structure. In both the Zn and Ni-treated roots, metal redistribution from x-ray beam induced damage is clearly evident in the areas where the slower tomograms were collected (upper arrows in Fig. 2a, d). Interestingly, in this case the damage was evidenced by a loss of Zn/Ni at the point of analysis which is in contrast with what observed when 3D tomography was attempted (Fig. 1). This different manifestation of beam damage deserves further investigation. There was no sign of beam damage for the shorter pixel transit time (lower arrows in Fig. 2a, d) either in terms of metal redistribution or mass loss. This indicates that we were able to beat the onset of radiation damage. Accordingly, the sinograms and tomographic reconstructions in Fig 2 are derived from the faster measurements.

The 2D distribution of Zn and Ni clearly differ and provide important information regarding the active areas of metal uptake (Fig. 2a, d). However a detailed understanding of the metal distributions across the roots is only possible from the reconstructed cross sections (Fig. 2c, f). This is because, due to the penetration of the x-ray beam, fluorescence is emitted from the whole volume in the path of the beam. Consequently the 2D image represents a planar compression of the root volume. The reconstructed cross sections show Ni accumulation mainly in the cortex and Zn localisation within distinct cells in the rhizodermis, outer cortex, and stele. This latter localisation could be due to high concentrations in xylem vessels. These reconstructions could suggest that the endodermis restricts the uptake of Ni more than it does Zn. This is in line with the metabolical requirement of these two metals. While adequate concentrations of Zn in plants are in the order of 10 to 100 µg/g, Ni requirements are much lower (generally below 1 µg/g). The information provided by the 2D map, however, suggests that the cross section distribution of Zn and Ni could also be caused by the differential pattern of Ni/Zn distribution in the root tip area (Fig. 2a, d). In fact, Zn seems to be accumulated at the root tip which could indicate a strong activity of Zn transporters in this area. From the root tip, Zn seems to be efficiently loaded in the xylem vessel for translocation to the shoot. In contrast, the concentrations of Ni at the root tip are much smaller indicating a more limited uptake in this region.

These observed distributions for Ni and Zn assist in elucidating the mechanisms of their rhizotoxicity. For Zn, there was substantial accumulation in the root apex with subsequent transport through the stele. Interestingly, excess Zn has previously been reported to have detrimental effects on both cell division and cell elongation in the root tip [17], [18]. Similarly, Rout and Das [19] reported that the major toxic effect observed in Zn-toxic plants occurs in the nuclei of root tip cells. The distribution of Zn observed in the present study would tend to support these hypotheses regarding the importance of the root apex in Zn toxicity. In contrast to Zn, Ni tended to accumulate mainly in cortical cells with reduced concentrations found in the stele (Figure 2). In a review of the literature, Chen et al. [20] suggested that Ni was likely toxic due to indirect effects, such as interference with nutrient uptake or possibly by inducing oxidative stress. Once again, the data from the present study regarding the distribution of Ni in fresh, hydrated roots would tend to support this hypothesis, although further work is clearly required. Interestingly, this observation regarding the distribution of Ni is similar to that reported by Seregin et al. [21] who studied several plant species exposed to toxic levels of Ni and reported that Ni often accumulates at the endodermis in non-hyperaccumulating plants. The 2D maps indicate that Zn uptake was greater than that of Ni, this result is in line with measured Zn concentrations reaching 1.1 µmol/g and Ni reaching 0.73 µmol/g (fresh root basis). These root tissue concentrations are similar to those reported elsewhere for other species grown in toxic solutions. For example, assuming cowpea roots are ca. 90% water (data not presented), these values compare well to those of 4.3 µmol/g (dry weight) for roots of bean (*Phaseolus vulgaris* L.) [22] and approximately 34 µmol/g (dry weight) for roots of maize (*Zea mays* L.) [23]. Similarly, roots of beans grown in solutions containing 13.5 µM Zn contained a Zn tissue concentration of 9 µmol/g (dry weight) [24].

Although the application of x-ray fluorescence microtomography to biological samples has been reported previously, this work shows the first example of this technique applied to hydrated specimens of extreme radiation sensitivity. It should be noted here that interesting alternative approaches based on the use of glass polycapillary half-lenses have been developed. These systems have been used to realize a confocal detection scheme, in fluorescence mode, able to provide 3D and internal 2D elemental mapping [25]. However, this specialized setup is not widely available and still needs to be assessed for hydrated biological samples.

The results reported here have been made possible by recent developments in detector technology that have increased detection efficiency by an order of magnitude and reduced per-pixel overheads to negligible levels. The collection of the faster 2D tomograms reported in Fig. 2 delivered approximately 90 kGy to the sample (estimated following [26]). Beam-induced metal redistribution was evident after a radiation dose of only 180 kGy. The extremely low level of this damage threshold is likely to be due to the high motility of metal(loid)s within the hydrated root. Recent high-resolution 3D fluorescence tomography of the diatom *Cyclotella meneghiniana* [27] reports an imaging dose of 50 MGy [12], almost 300 times the present work. Use of the Maia detector for imaging *Cyclotella* would enable the imaging dose to be reduced by a factor of 30–50 to around 1 MGy. It is evident that the use of this new generation of fast detectors is essential for performing analysis on highly hydrated biological samples before the onset of significant beam damage and dehydration. Indeed, fast detection is vital for the minimisation of motion artifacts when imaging this type of sample. With further developments in detector performance [12] it can be envisaged that *in vivo* applications of x-ray fluorescence microtomography for biological samples will provide a powerful spectroscopic tool complementing *in situ* molecular biology imaging techniques.

## Materials and Methods

### Plant growth

Seeds of cowpea (*Vigna unguiculata* (L.) Walp. cv. white Caloona) were germinated in rolls of paper towel placed vertically in tap water for 3 d. During this germination period, the seeds were transported to the Australian Synchrotron, Melbourne, Australia. Glass beakers (600 mL) were placed into a water bath heated to 26°C and filled to the brim with 650 mL of 1 mM CaCl_2_ and 5 µM H_3_BO_3_. Perspex strips, each with seven seedlings, were placed on top of the beakers and the seedlings grown for 12 to 18 h in order to acclimatise. The seedlings were then transferred to other beakers which contained the metal of interest in addition to the basal 1 mM CaCl_2_ and 5 µM H_3_BO_3_. Two metals were investigated, these being 5 µM Ni and 40 µM Zn (both for 24 h exposure). These concentrations have been shown to reduce growth by ca. 70 to 90% for a 48-h exposure period [28]. The metals were added using appropriate volumes of a 6.5 mM stock solution (NiCl_2_.6H_2_O or ZnSO_4_.7H_2_O). All solutions were continuously aerated. The pH of the nutrient solutions was not adjusted, although the average value upon harvest (5.4) was slightly lower than upon mixing (5.6). Modelling with GeoChem-EZ [29] indicated that the free ion (i.e. Ni^2+^ or Zn^2+^) accounted for ca. ≥98% of the total Ni or Zn in all solutions.

To allow assessment of the bulk metal concentration in the apical tissues of the roots, seedlings were grown for 24 h in Ni- or Zn-containing solutions. For each treatment, ca. 50 seedlings were harvested, and their root apices (ca. 150 mg fresh mass) digested using 5∶1 nitric/perchloric acid, with Merck extra pure nitric acid. The roots were placed into 5 mL volumetric flasks and 1 mL of acid added. Following digestion, the samples were diluted to 5 mL using deionized water before analysis by inductively coupled plasma mass spectrometry.

### Tomographic setup

Synchrotron-based x-ray fluorescence microtomography was performed at the X-ray Fluorescence Microscopy (XFM) beamline at the Australian Synchrotron [30]. This beamline uses an in-vacuum undulator to produce a brilliant x-ray beam from which the 15.6 keV x-rays were selected using a Si(111) monochromator. A Kirkpatrick-Baez mirror pair was used to focus the beam to a spot slightly smaller than 2×2 µm. Specimen translations were performed using a pair of crossed linear stages. Roots were mounted (inside their capillaries, for the tomographic measurements) onto a pin which was in turn attached to a small pair of stages that were used to bring the root to the rotation centre. Rotation was achieved using a 200 step/rev stepper motor in full-step mode.

Elemental maps were collected using the Maia detector system. The Maia detector uses an annular array of 384 1-mm^2^ silicon-diode detectors positioned in the backscatter geometry so as to subtend a very large 1.3-steradian solid-angle and to achieve high count-rate capacity [14]. The Maia detector is backed up by a massively-parallel FPGA processor which can perform real-time analysis to output a stream of x-ray events characterised by x-ray energy, time-over-threshold, and detector identity. This x-ray event-mode stream joins with a pixel event-mode stream which is used to identify the locations of the pixel boundaries in the scanned images. Acquisition with this system is essentially overhead-free, enabling routine acquisition on the fly with pixel dwell times down to 50 µsec.

### Tomographic analysis

Root samples, prepared as described above, were harvested, quickly rinsed, and inserted in a polyimide capillary partially filled with water. The capillary (internal diameter 860 µm, wall thickness 25 µm) was sealed with wax in order to create a moist chamber, and immediately mounted and centred on the rotation stage. The analyses were conducted at a distance approximately 1.5 to 2.0 mm from the root tip.

3D tomograms were acquired by collecting 2D micro x-ray fluorescence (µ-XRF) maps over 100 angles spaced over 360°. The area mapped extended to 40 µm in the vertical direction and 1.5 mm in the horizontal axis. A full 360-degree range was chosen to probe self-absorption effects, but measurements over the second half of the range were made unique by offsetting them by 1.8-degrees relative to the first (i.e., measurements were made at 3.6-degree intervals over two separate ranges, from 0–180 degrees and from 181.8–361.8 degrees). The sampling interval was 2×2 µm with a transit time of 2.6 ms.

2D tomograms were acquired by scanning a single transect in order to obtain µ-XRF line scans over 200 angles (from 0° to 360°). A full rotation was used so as to enable assessment of beam damage and self absorption by the specimen. The sampling interval was 2 µm and two transit times were tested on each root (7.8 ms and 3.9 ms, with corresponding acquisition times of about 14 and 9 min). At the end of the 2D tomograms, µ-XRF maps were collected over an area extending 2.5 mm from the root tip in order to assess whether beam damage had occurred. In this case the sampling interval was 2×2 µm with a transit time of 3.9 ms.

The XRF events were analysed using GeoPIXE [31], [32] which employs the Dynamic Analysis algorithm to remove backgrounds and resolve overlapping peaks to generate a rotation series of projected elemental maps. Self absorption effects were found to be insignificant by direct observation of the various elemental sinograms. This result comes as no surprise, as the absorption lengths in water for Cu and Zn Kα-fluorescence are around 1 mm. Had there been significant distributions of lighter elements such as K and Ca, however, self-absorption corrections would have been required, as the relevant attenuation length drops below 70 µm. The elemental projections were aligned by using the strong, unambiguous Compton scattering signal produced by the capillary. Tomographic reconstructions were performed using an implementation of the GridRec algorithm (http://cars9.uchicago.edu/software/idl/tomography.html) interfaced with the IDL programming language (http://www.ittvis.com/). Spatial resolution is estimated to be around 4 µm (2 pixels) due to limitations in the projection alignment process.

## Supporting Information

Movie S1 Zinc (magenta) x-ray fluorescence maps of *V. unguiculata* root volume collected at 100 orientations. The Compton signal is in green.
(WMV)

Movie S2 Tomographic reconstructions of Zn (magenta) distribution in a *V. unguiculata* root. The Compton signal is in green. Twenty consecutive virtual cross sections are presented.
(WMV)
